# Effect of powered circular stapler in colorectal anastomosis after left-sided colic resection: systematic review and meta-analysis

**DOI:** 10.1007/s00384-024-04729-1

**Published:** 2024-09-27

**Authors:** Andrea Scardino, Carlo Galdino Riva, Luca Sorrentino, Sara Lauricella, Alberto Aiolfi, Matteo Rottoli, Gianluca Bonitta, Marco Vitellaro, Luigi Bonavina, Davide Bona, Michael Kelly, Emanuele Rausa

**Affiliations:** 1 Colorectal Surgery Unit, Fondazione IRCCS Istituto Nazionale Dei Tumori, Via Venezian 1, 20133 Milan, Italy; 2 General Surgery Residency Program, University of Milan, Milan, Italy; 3 Department of Biomedical Science for Health, Division of General Surgery, University of Milan, Istituto Clinico Sant’Ambrogio, Milan, Italy; 4 Surgery of the Alimentary Tract, IRCCS Azienda Ospedaliero Universitaria Di Bologna, Bologna, Italy; 5 Department of Biomedical Sciences for Health, Division of General Surgery, IRCCS Policlinico San Donato, University of Milan Medical School, Milan, Italy; 6 School of Medicine, Trinity College Dublin, Dublin, Ireland; 7 The Trinity St. James’s Cancer Institute, Dublin, Ireland

**Keywords:** Powered circular stapler, Colorectal surgery, Anastomotic leak, Left-sided colonic resection, Left-sided anastomosis

## Abstract

**Purpose:**

Anastomotic leak (AL) remains the most important complication after left-sided colic anastomoses and technical complications during anastomotic construction are responsible of higher leakage incidence. Powered circular stapler (PCS) in colorectal surgery has been introduced in order to reduce technical errors and post-operative complications due to the manual circular stapler (MCS).

**Methods:**

A systematic review and meta-analysis were performed. An electronic systematic search was performed using Web of Science, PubMed, and Embase of studies comparing PCS and MCS. The incidence of AL, anastomotic bleeding (AB), conversion, and reoperation were assessed. PROSPERO Registration Number: CRD42024512644.

**Results:**

Five observational studies were eligible for inclusion reporting on 2379 patients. The estimated pooled Risk Ratios for AL and AB rates following PCS were significantly lower than those observed with MCS (0.44 and 0.23, respectively; both with *p* < 0.01). Conversion and reoperation rate did not show any significant difference: 0.41 (95% CI 0.09–1.88; *p* = 0.25) and 0.78 (95% CI 0.33–1.84; *p* = 0.57); respectively.

**Conclusion:**

The use of PCS demonstrates a lower incidence of AL and AB compared to MCS but does not exhibit a discernible influence on reintervention or conversion rates. The call for future randomized clinical trials aims to definitively clarify these issues and contribute to further advancements in refining surgical strategies for left-sided colonic resection.

**Supplementary Information:**

The online version contains supplementary material available at 10.1007/s00384-024-04729-1.

## Introduction

Colorectal cancer (CRC) is the most prevalent tumor of the gastroenteric system worldwide with an incidence of one million new cases annually [1–3]. Specifically, about 35% of CRC manifest in the rectum. The management of rectal cancer has profoundly changed over the last two decades and is currently based on the concept of multimodal therapy. This comprehensive approach includes neoadjuvant radiochemotherapy (RCT), surgery and adjuvant chemotherapy that resulted in a notable decrease in the rate of local tumor recurrence [4]. Concurrently, diagnostic modalities have experienced a profound transformation, leading to earlier and more accurate disease detection. In this context, it is fundamental to precisely determine the loco-regional extension of the tumor throughout a magnetic resonance imaging and endorectal ultrasound in order to allocate the patients to the most appropriate treatment.

Notably, rectal surgery is burdened by a remarkable risk of anastomotic leak (AL), ranging from 5 to 20%, and affects morbidity, mortality and long-term oncological outcomes [5–7]. Some studies investigated the risk factors for AL and found that additionally to intraoperative causes (i.e., duration of surgery, intraoperative contaminations, tension at the anastomotic site, blood supply and number of cartridges used to close the rectal stump) [3, 8], some variables were non-adjustable (i.e., sex, age, history of RT, ASA score, patient’s co-morbidities and tumor location) and others potentially adjustable (i.e., malnutrition, smoking, obesity) [9]. Typically, colorectal anastomoses are performed in a double stapling technique (DST) which was developed by Knight-Griffen in 1980 [10] and standardized by Cohen in 1984 [11]. The conventional approach to colorectal anastomoses involves the use of manual circular staplers (MCS), which are considered the standard of care for surgical reconstruction. However, the effectiveness of these staplers depends on the consistent and stable application by surgeons and all manual circular staplers were fired under manual grip force, with high incidence of technical complications [12]. In 2019, the first powered circular stapler (PCS), the Ethicon® Circular Powered Stapler, was released to reduce technical errors and post-operative complications [13]. The practical application of the PCS has been associated with improved clinical outcomes and fewer technical issues in the creation of left-sided anastomoses [14–19]. The aim of this systematic review and meta-analysis was to quantitatively investigate and compared the short-term outcomes of PCS versus MCS.

## Materials and methods

### Search strategy

This systematic review aimed to evaluate studies on colorectal anastomosis performed with a powered circular stapler (PCS). The Preferred Reporting Items for Systematic Reviews and Meta-analyses (PRISMA) checklist [20] and MOOSE guidelines [21] ensured adherence to rigorous reporting standards. Since the review analyzed existing data, ethical approval was not required.

Two independent reviewers (CGR and ER) conducted a comprehensive literature search across Web of Science, PubMed, and Embase. They searched for English-language publications by combining the following terms: “powered circular stapler”, “circular stapler”, “Knight-Griffen”, “double stapling technique”, “colorectal surgery”, “colorectal disease,” “rectal resection,” “left-sided anastomosis”, “left-sided colorectal resections”, “low anterior resection”, “rectal anastomosis” and “colorectal cancer” using “AND” and “OR”. Additionally, reference lists of identified articles were reviewed for further relevant studies. The study protocol was registered on PROSPERO (Registration Number: CRD42024512644).

### Inclusion and exclusion criteria

Inclusion criteria: (a) articles comparing surgical outcomes for left-sided anastomoses performed with PCS and MCS; (b) English written; (c) papers with the longest follow-up or the largest sample size in case of articles published based on the same data set. Exclusion criteria: (a) not English-written; (b) no clear methodology; (c) articles not reporting any of the a priori defined primary outcomes; (d) articles with less than 10 patients. Given the potential biases and inconsistencies inherent in gray literature, we prioritized peer-reviewed sources for our research.

### Data extraction

Two authors (AS and CGR) independently extracted data from eligible studies. Data extracted included study characteristics (first author name, year, and journal of publication), number of patients included in the series, time frame, clinical and demographic characteristics of patients’ population, type of surgical procedure, and postoperative outcomes. Disagreements between authors were resolved by consensus; if no agreement could be reached, a third senior author (ER) made the decision.

### Quality assessment

Two investigators (AS and CGR) independently assessed the methodological quality of the enrolled papers using the Risk of Bias In Non-randomized Studies — of Interventions (ROBINS-I) tool [22], which categorizes risk of bias as low, moderate, serious, critical and unclear, with the risk of bias category for each study being reported. If a study’s risk of bias was categorized as serious, critical or unclear, the effect of removing this study was tested and the relevant outcomes reported (Table 1).

**Table 1:**
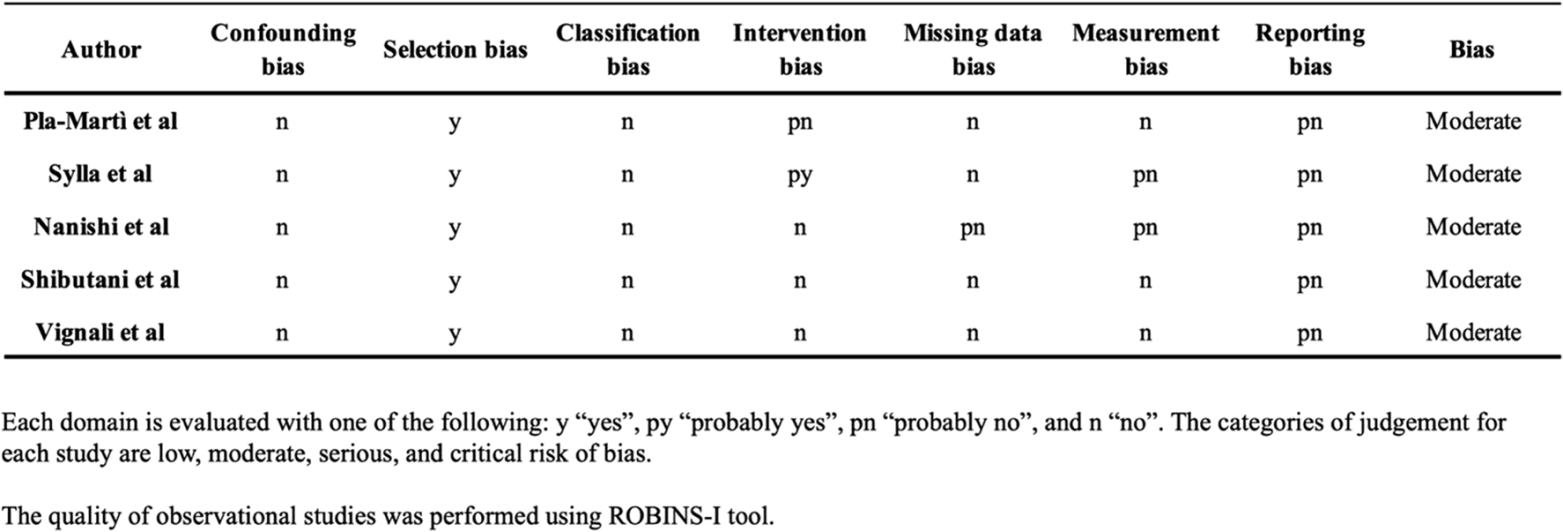
Quality assessment of the included studies

### Outcomes of interest

The primary outcome was the rate of the AL. The secondary outcomes were the anastomotic bleeding (AB), reoperation and conversion rate.

### Statistical analysis

The findings of this study were synthesized using frequentist random-effect meta-analysis, aggregating risk ratios (RR). The DerSimonian–Laird estimator was employed to determine the variance of the true effect size (τ2), alongside an inverse-variance method [23, 24]. Heterogeneity across studies was assessed through the I2 index and Cochran’s Q test [25]. Levels of statistical heterogeneity were categorized as low, moderate, and high for I2 values of 25, 50, and 75%, respectively, with significance set at *p* < 0.10 [26, 27]. Confidence intervals (CI) at 95% were computed using the Wald-type method for pooled measurements, while the Clopper-Pearson method was applied for individual intervals. The 95% CI for the I2 index followed Higgins and Thompson’s approach [28]. Prediction intervals for the treatment effect of new studies were calculated based on Borenstein et al. [25]. Sensitivity analysis was conducted by iteratively excluding one study at a time to ensure the robustness of the overall results, considering varying sample sizes across studies. Statistical significance was defined as a two-sided *p* value of less than 0.05. All analyses and visualizations were performed using R software version 3.2.2 [29]. Consistent with Cochrane guidelines, publication bias assessment was not conducted due to the inclusion of fewer than 10 studies per data comparison in our search.

## Results

### Literature search and study characteristics

**Fig. 1:**
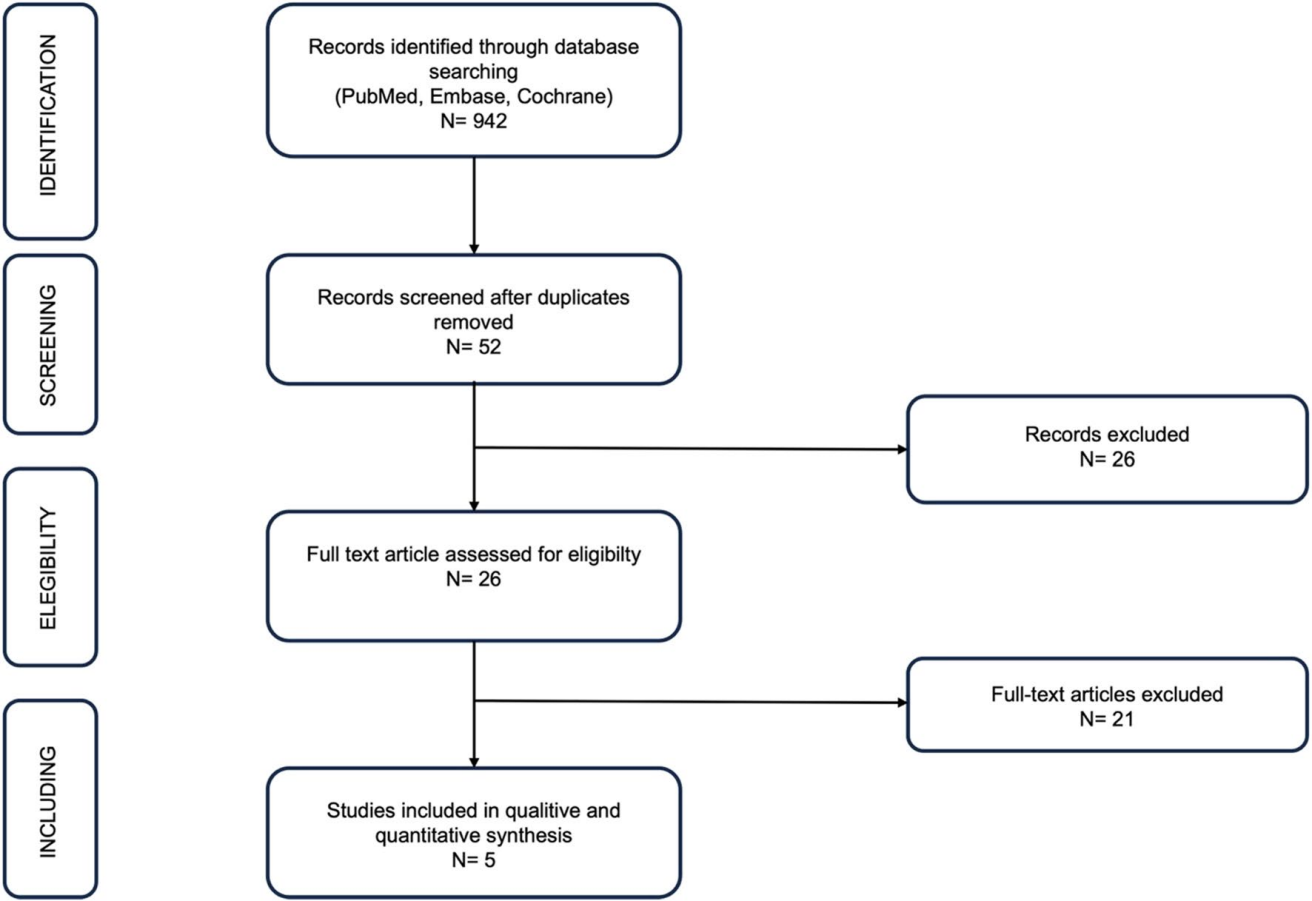
Flowchart of studies retrieved from literature search

The electronic search yielded a total of 942 publications that met the specified criteria outlined above. Following the removal of duplicate entries, 52 publications were subjected to a thorough examination. Subsequent screening revealed that only 5 articles satisfied the predetermined inclusion criteria [14, 15, 17–19], as depicted in Fig. 1.

### Patient characteristics

A total of 2379 patients were included in this analysis, of whom 580 (24.4%) underwent to anastomosis with PCS and 1799 patients (75.6%) with MCS. Gender breakdown was 55.7% male (*n* = 1326) and 44.3% female (*n* = 1053). Median age, specified in 3 studies, ranged from 66 to 69. Preoperative BMI, reported in 2 studies, ranged from 21.2 to 26.7 (Table 2a).

**Table 2:**
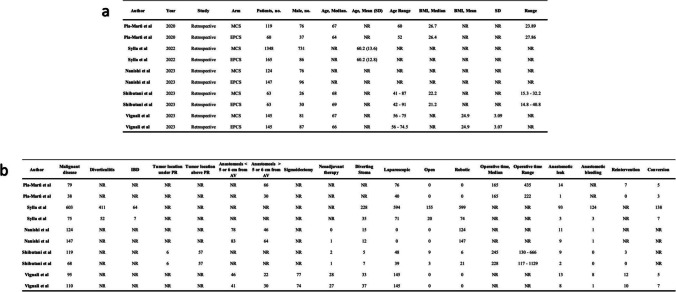
List of included studies with (a) demographic and (b) clinical characteristics of patients

A total of 1458 patients (61.3%) had surgery for malignancy and 3 studies outlined that 59 patients (8.6%) received neoadjuvant chemotherapy. Stratifying the population for staging was not doable because these data in enrolled studies are lacking. The location of the anastomosis was defined in 3 studies: the anastomoses were performed above 5 cm from the anal verge in 409 patients (55.3%) and below 5 cm from the anal verge in 248 (44.2%). Diverting stoma was performed in 327 patients (16.9%). The colonic resection was carried out laparoscopically in 1158 cases (48.7%), robotically in 971 (40.9%) and open in 187 (7.9%) (Table 2b).

### Definitions of AL across the enrolled studies

Pla‑Marti, Nanishi and Vignali et al. defined AL according to the International Study Group definition of rectal cancer [30]. Shibutani et al. assessed AL as the extravasation observed on radiologic examinations. Sylla et al. codified postoperative complications according with the corresponding International Classification of Diseases, 10th Revision, Clinical Modification and Procedure Classification System (ICD-10-CM/ICD-10-PCS) diagnosis and procedure codes. As no specific diagnosis code is available for AL in the ICD-10-CM taxonomy, AL diagnoses were defined following the coding conventions of Kang et al. [31].

### Anastomotic leakage rate

**Fig. 2:**
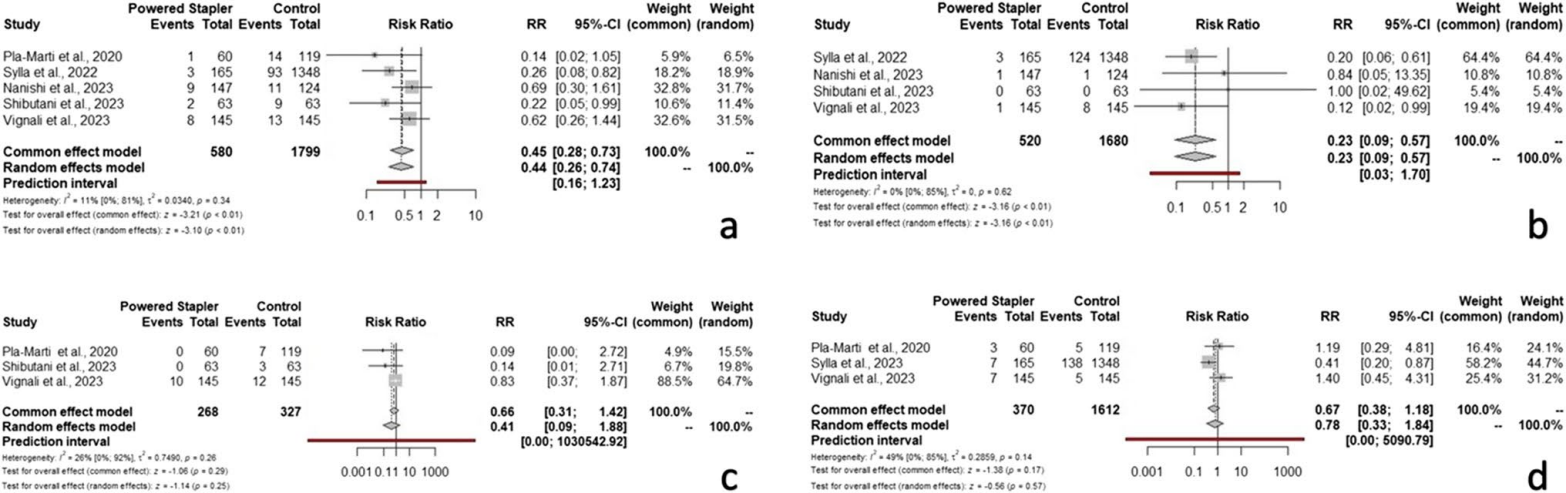
Forest plots of the meta-analysis estimates the risk ratio for (**a**) anastomotic leak (AL) rate; (**b**) anastomotic bleeding (AB) rate; (**c**) reoperation rate; (**d**) conversion rate

All included studies involving 2379 patients were analyzed using a random-effects model. The analysis revealed that PCS treatment significantly reduced the AL rate compared to the MCS. The estimated pooled Risk Ratio is 0.44 (95% CI 0.26–0.74; *p* < 0.01). The combination of data from all studies results in low heterogeneity (I2 = 11%, 95% CI 0.0–81%; *p* = 0.34) and τ2 = 0.034 (Fig. 2a).

### Anastomotic bleeding rate

Four included studies involving a total of 2200 patients, reported AB and were included for this meta-analysis. Considering random effect model, the estimated pooled Risk Ratio of AB is 0.23 (95% CI 0.09–0.57; *p* < 0.01). The prediction interval is 0.03–1.70. The heterogeneity is not significant (I2 = 0%, 95% CI 0.0–85%; *p* = 0.62) and τ2 = 0 (Fig. 2b).

### Reoperation rate

Three studies which include a total of 595 patients, reported reoperation rate (RR) and were included for this meta-analysis. Considering random effect models, the estimated pooled Risk Ratio of RR is 0.41 (95% CI 0.09–1.88; *p* = 0.25). The prediction interval is 0.0–1030542.92. The heterogeneity is low (I2 = 26%, 95% CI 0.0–92%; *p* = 0.26) and τ2 = 0.74 (Fig. 2c).

### Conversion rate

Three studies which include a total of 1982 patients, reported conversion rate (CR) and were included for this meta-analysis. Considering random effect models, the estimated pooled Risk Ratio of CR, is 0.78 (95% CI 0.33–1.84; *p* = 0.57). The prediction interval is 0.0–5090.79. The heterogeneity is moderate (I2 = 49%, 95% CI 0.0–85%; *p* = 0.14) and τ2 = 0.28 (Fig. 2d).

## Discussion

This study revealed that the use of PCS was associated with a reduced occurrence of AL and AB when compared to MCS. However, the choice between PCS and MCS did not exhibit any discernible impact on the rates of reoperation or conversion. The multimodal treatment strategy (MTS) employed for low rectal cancer introduces both technical and oncological challenges, as the goal is to enhance oncological outcomes while minimizing both short and long-term surgical consequences through minimally invasive techniques. Particularly, the adoption of a MTS for rectal cancer, including neoadjuvant chemoradiotherapy allows for tumor down-staging, with potential complete response rates of approximately 18% and a possible increasing in sphincter preservation rate [32–34].

Traditionally, abdominoperineal resection (APR) served as the primary surgical approach for both low rectal cancer and anal cancer [35]. However, contemporary surgical management, influenced by the evolution of anterior resection techniques such as mechanical staplers, updates in anastomotic techniques, and transanal total mesorectal excision (TaTME), has led to a shift, relegating APR predominantly to cases of anal cancer [36, 37]. Regarding updates in rectal surgery, the introduction of the DST has enabled safe and rapid anastomosis. However, multiple uses of the cartridge of a linear stapler for rectal transection remain a risk factor for AL. The single stapling technique has gained attention as an alternative, with other modified techniques like “planned” DST, suprapubic port for staple firing, and lateral invagination technique for “dog ear” prevention [38]. A prospective multicenter observational study to evaluate the efficacy and safety of the reinforcement cartridge for AL (trial ID: UMIN000029543) is also underway in Japan, and the results of this study are awaited [39]. Moreover, the TaTME has emerged to address challenges in difficult pelvic dissections associated with rectal cancer [36–38]. Notably, it facilitates various anastomotic techniques without the necessity for trans-abdominal rectal transection.

Concerning intraoperative setting, surgeons have developed various AL tests to promptly detect defects and reduce the incidence. Air leak testing, endoscopic examination and indocyanine green (ICG) have been validated; recent network meta-analysis demonstrating the potential of ICG to significantly reduce postoperative AL rates [40].

Despite advancements in operative and postoperative care, AL still represents a substantial postoperative issue, occurring in approximately 20% of patients following rectal resection. This complication heightens mortality and has a negative impact on survival. Moreover, the majority of patients experiencing symptomatic AL ultimately require a permanent stoma; which consistently diminishes quality of life. The clinical presentation of AL varies widely, ranging from concealed AL to severe sepsis, and the extent to which this correlates with spectrum of treatment strategies which vary from conservative management and reintervention [41, 42]. In our analysis, a diverting stoma was performed in 372 patients (15.4%) at primary surgery. These data reinforce the hypothesis that diverting stoma does not reduce leak rate [43]. A recent study indicated that diverting stomas is unnecessary in approximately 80 to 95% of patients and may uniquely mitigate the severe repercussions induced by AL [44]. As the matter of the fact, these evidences challenge the conventional belief that diverting stomas reduce AL rates, emphasizing their potential unnecessary use in a significant percentage of patients without AL. Additionally, diverting stomas increase the risk of stoma-related complications and the need for a second operation.

The novel PCS introduces design improvements in circular stapling technology, addressing potential issues with manual circular staplers, such as the force required for anastomosis leading to unwanted movements and microvascular trauma. Powered firing, 3D stapler configuration, and Gripping Surface Technology contribute to improved stability, ergonomic design, and precise compression, promoting optimal conditions for anastomotic healing [13, 45].

A potential limitation of the study arises from the fact that the majority of the selected articles fail to specify whether two- or three-staple line circular staplers were employed. These specific data were only outlined in the study by Nanishi et al., where 111 out of 147 patients underwent surgery using a two-row manual circular stapler [15]. On the other side, the recent findings of Wang et al. showed no significant difference in anastomotic complications between two-row and three-row manual circular staplers [46].

In terms of anastomotic bleeding outcomes, the meta-analysis indicates that the use of PCS reduces the risk of suture bleeding by over 20% compared to MCS for left-sided anastomoses. It can be speculated the distribution of compression throughout the anastomosis may be a contributing factor to this result, promoting greater reproducibility of the procedure, maintaining a stable position, and employing a gentler approach to the tissue.

Regarding reoperation and conversion rates, the meta-analysis did not demonstrate significant superiority of PCS over MCS, attributing this to moderate heterogeneity and a wide range of prediction intervals. The study emphasizes the increasing conservative management in subclinical AL, negating the need for reintervention. Rottoli et al. showed the association between favorable postoperative and pathological outcomes and colorectal surgery in high-volume centers. Specifically, the conservative management has implemented the spectrum of non-operative possibilities passing by the antibiotics to percutaneous or transanal drainage [47].

The clinically impactful surgical complications examined, specifically AL and AB, have substantial economic consequences in terms of costs, length of stay, and the risk of hospital readmission [48]. Acknowledging the limitations of the meta-analysis, including the lack of high-quality evidence due to the predominance of cohort studies, the research represents a considerable sample size of 2379 surgical patients from different continents. Moreover, the study was planned in agreements with PRISMA 2020 guidelines, and followed a pre-defined methodology that was expressed in PROSPERO. This included comprehensive outcome measures and the evaluation of quality at study level (risk of bias).

Last, the AL was not objectively defined or graded using an international score across the enrolled studies. Present literature indicates various definitions of AL, which can vary based on patients’ clinical symptoms, as well as endoscopic or radiological findings. This diversity impedes straightforward comparison of study results and consequently complicates drawing definitive conclusions regarding preferred operative and perioperative management strategies in routine clinical practice. However, the selection criteria appear to have led to a homogeneous study population, as evidenced by low heterogeneity. These strengths position this study as a relevant resource for planning future randomized clinical trials that directly compare PCS to MCS.

In conclusion, the use of PCS demonstrates a lower incidence of AL and AB compared to MCS but does not exhibit a discernible influence on reintervention or conversion rates. The call for future randomized clinical trials aims to definitively clarify these issues and contribute to further advancements in refining surgical strategies for left-sided colonic resections.

## Supplementary information

Below is the link to the electronic supplementary material.

Supplementary file1 (PDF 203 KB)

Supplementary file2 (PDF 7.35 KB)

## Data Availability

Data supporting research will be made available upon request to the corresponding author.
