# Supplementary material for: Effect of powered circular stapler in colorectal anastomosis after left-sided colic resection: systematic review and meta-analysis
Source: Int J Colorectal Dis. 2024 Sep 27;39(1):152. doi: 10.1007/s00384-024-04729-1 (PMC11436432; doi:10.1007/s00384-024-04729-1)
Supplement: Supplementary file 2 — Supplementary file2 (PDF 7.35 KB) [file 384_2024_4729_MOESM2_ESM.pdf]

MOOSE Guidelines for Meta-Analyses and Systematic Reviews of Observational Studies*

Title — Identify the study as a meta-analysis (or systematic review)

Abstract — Use the journal's structured format

Introduction

Present
- The clinical problem
- The hypothesis
- A statement of objectives that includes the study population, the condition of interest, the exposure or intervention, and the outcome(s) considered

Sources

Describe
- Qualifications of searchers (eg, librarians and investigators)
- Search strategy, including time period included in the synthesis and keywords
- Effort to include all available studies, including contact with authors
- Databases and registries searched
- Search software used, name and version, including special features used (eg, explosion)
- Use of hand searching (eg, reference lists of obtained articles)
- List of citations located and those excluded, including justification
- Method of addressing articles published in languages other than English
- Method of handling abstracts and unpublished studies
- Description of any contact with authors

Study Selection

Describe
- Types of study designs considered
- Relevance or appropriateness of studies gathered for assessing the hypothesis to be tested
- Rationale for the selection and coding of data (eg, sound clinical principles or convenience)
- Documentation of how data were classified and coded (eg, multiple raters, blinding, and interrater reliability)
- Assessment of confounding (eg, comparability of cases and controls in studies where appropriate)
- Assessment of study quality, including blinding of quality assessors; stratification or regression on possible predictors of study results
- Assessment of heterogeneity
- Statistical methods (eg, complete description of fixed or random effects models, justification of whether the chosen models account for predictors of study results, dose-response models, or cumulative meta-analysis) in sufficient detail to be replicated

Results

Present
- A graph summarizing individual study estimates and the overall estimate
- A table giving descriptive information for each included study
- Results of sensitivity testing (eg, subgroup analysis)
- Indication of statistical uncertainty of findings

Discussion

Discuss
- Strengths and weaknesses
- Potential biases in the review process (eg, publication bias)
- Justification for exclusion (eg, exclusion of non–English-language citations)
- Assessment of quality of included studies
- Consideration of alternative explanations for observed results
- Generalization of the conclusions (ie, appropriate for the data presented and within the domain of the literature review)
- Guidelines for future research
- Disclosure of funding source

*Modified from Stroup DF, Berlin JA, Morton SC, Olkin I, Williamson GD, Rennie D, et al. Meta-analysis of observational studies in epidemiology: a proposal for reporting. Meta-analysis Of Observational Studies in Epidemiology (MOOSE) group. JAMA 2000;283:2008–12. Copyrighted © 2000, American Medical Association. All rights reserved.
